# Can Religious and Spiritual Struggle Enhance Well-Being? Exploring the Mediating Effects of Internal Dialogues

**DOI:** 10.1007/s10943-018-00755-w

**Published:** 2019-01-02

**Authors:** Beata Zarzycka, Małgorzata M. Puchalska-Wasyl

**Affiliations:** Institute of Psychology, The John Paul II Catholic University of Lublin, Al. Racławickie 14, 20-950 Lublin, Poland

**Keywords:** Religious/spiritual struggle, Internal dialogues, Well-being

## Abstract

Although a number of studies have demonstrated links between religious/spiritual struggle and well-being, only a few have examined what makes religious/spiritual struggle increase or decrease well-being. The present paper aims to examine different forms of internal dialogues (IDs) as potential mediators of the relationship between religious/spiritual struggle and well-being among Roman Catholics. There were 143 respondents (81 female) who participated in the study. We applied the Religious and Spiritual Struggle Scale, the Internal Dialogical Activity Scale, and the Psychological Well-Being Scale. The results showed that religious/spiritual struggle triggers IDs. Three types of IDs—ruminative, supportive, and social simulation dialogues—were mediators in the relationship between religious/spiritual struggle and well-being. Although religious/spiritual struggle decreased well-being through its effect on ruminative dialogues, it improved well-being through its effects on supportive and social simulation dialogues.

## Introduction

People turn to religion to receive consolation or provide meaning to their life (Park 2005a, b). Religion can be an important predictor of health and well-being (Krok 2015, 2016; Park and Slattery 2013; Zarzycka and Tychmanowicz 2015). The beneficial effects of religion on mental health have been established over the past decades (for reviews, see George et al. 2002; Hill and Pargament 2003; Koenig et al. 2001; Larson and Larson 2003).

However, thinking about God does not necessarily bring only comfort. When people try to live according to their religious beliefs but cannot live up to them, they feel guilty. Some people feel offended by other believers, for example, when they witness hypocrisy by religious people (Krause et al. 2000). Psychologists coined the term “religious and spiritual struggle” to recognize different forms of distress or conflicts in the religious or spiritual realm (Exline 2013; Exline and Rose 2013). Religious and spiritual struggle can focus on several domains, categorized as supernatural, intrapersonal, and interpersonal (Pargament et al. 2005). The supernatural struggle can refer directly to God (divine) and be expressed as negative emotions or conflicts related to beliefs about God, or they can refer to spiritual forces of evil (demonic), including a belief that people are subject to the attacks of evil spirits. Intrapersonal struggle can manifest as the tension people experience between the virtues they espouse and their actual behavior (moral), doubts about religion, or losing one’s sense of ultimate meaning in life. Interpersonal struggle involves religious conflicts between an individual and congregation members, clergy, or members of other religious groups (Zarzycka and Zietek 2018).

Most studies suggest that religious struggle worsens health (e.g., Ano and Vasconcelles 2005; Exline and Rose 2013; Smith et al. 2003). A meta-analysis of 49 studies reported positive correlations between religious/spiritual struggle and anxiety, depression, negative mood, a sense of guilt, and social dysfunctions (Ano and Vasconcelles 2005). Similar relationships were reported in a meta-analysis of 147 studies (Smith et al. 2003). However, some researchers claim that people can benefit from their religious/spiritual struggle. This idea refers to psychological concepts that assume that individual growth can happen through suffering, and crisis can be a transition point between the life stages (e.g., Erikson 1968). The research has showed that religious/spiritual struggle can be associated with such positive outcomes as stress-related growth, spiritual growth, open-mindedness, self-actualization, and lower levels of prejudice (Hill and Pargament 2003; Zarzycka and Zietek 2018). However, only a few studies have been conducted so far to assess how religious/spiritual struggle is associated with positive outcomes. Pargament et al. (2006) showed that religious involvement, the ability to cope with adverse situations, and the severity of religious/spiritual struggle are moderators in the relationship between this kind of struggle and individual growth. Zarzycka and Zietek (2018) demonstrated that religious/spiritual struggle can enhance satisfaction with life and reduce anxiety through its effect on spiritual growth and meaning making. In this study, we aimed to explore further how religious/spiritual struggle can lead to positive outcomes. We assumed that internal dialogues can be mediators in the relationship between religious/spiritual struggle and well-being.

The concept of internal dialogue (ID) is strongly rooted in the dialogical self theory (Hermans 2003; Hermans and Gieser 2012; Hermans and Hermans-Jansen 1995), according to which a dialogical relationship exists not only between the self and others (interpersonal dialogues) but also within the self (intrapersonal/internal dialogues). We assume that a person is engaged in an ID when he/she alternately adopts (at least) two different viewpoints, and the utterances formulated from these viewpoints (silently or aloud) respond to one another (Puchalska-Wasyl 2016a, b). According to the dialogical self theory, the viewpoints adopted in IDs can represent personal perspectives (e.g., “I as a believer,” “I as a doubter,” “I as one deprived of meaning in life”) and/or someone else’s perspectives (e.g., God’s viewpoint, a religious person’s perspective, a religious institution’s position).

Difficult situations are usually a starting point for an ID because people are often working through their internal dilemmas and interpersonal conflicts by means of IDs (Puchalska-Wasyl 2016a, b, c, 2017). Analyzing 649 internal interlocutors, Puchalska-Wasyl (2006, 2016a, c) has identified seven groups of functions fulfilled by an ID, which have been called meta-functions or key functions. They are as follows: support, substitution, exploration, bond, self-improvement, insight, and self-guiding. Key function of support concerns such situations as when an ID is a source of hope, a sense of security, and a source of meaning in life. When an ID is a substitute for a real contact, when it is a form of argumentation practice or catharsis, then one can say it performs a substitution function. Sometimes people search for new experiences or escape from dull reality by means of IDs; in that case, IDs fulfill an exploration function. A bond function means that IDs provide the experience of a deep relation with someone close and a sense of being needed. IDs can perform a self-improvement function when they are a form of warning against a mistake or learning from other people’s mistakes, or when they facilitate formulating self-evaluation criteria. An insight function is fulfilled by IDs that are a way of gaining a new perspective, advice, and distance from a problem. Finally, self-guiding means that an ID is conducted in such a way that it motivates the person to develop, promotes setting new goals, or helps to acquire a sense of control over the situation. Studies by Hermans and Hermans-Jansen (1995) and Hermans (2003) have shown that voicing opposing viewpoints regarding a problem is conducive to well-being and more adaptive psychological functioning. Additionally, an integration of all the viewpoints involved in an ID enhances situational self-esteem and positive emotions (Borawski 2011) as well as diminishes discrepancies between the ideal self and ought self (Młynarczyk 2011). Moreover, Cieślar (2015) has proved that imaginary dialogue is an important way to deal with stress in an adverse situation, more effective than creating a list of potential solutions. Taking all these positive functions into account, it can be assumed that IDs will strengthen when experiencing religious/spiritual struggle that is a form of distress or conflicts referring to the religious or spiritual realm.

In this context, we decided to test different forms of IDs as potential mediators of the relationship between religious/spiritual struggle and well-being. We took into account the following types of ID: pure dialogical activity, identity, supportive, ruminative, confronting, social simulation, and perspective-taking dialogues (this classification will be presented in detail further—see Measures). Because our study was exploratory one, we did not advance any hypotheses. Instead, we posed some questions based on the knowledge of different types of internal dialogues and religious/spiritual struggle. Do all the types of internal dialogues relate to religious/spiritual struggle or are there some specific forms of internal dialogues that relate to religious/spiritual struggle and mediate the effect of struggle on well-being? Can religious/spiritual struggle improve well-being through its effects on IDs or not? In this case, we predicted that religious struggle could improve well-being through its effect on all the given types of internal dialogues except for ruminative dialogue, in which people invoke difficult topics in their own minds and delve into them. Therefore, we expected that ruminative dialogue could strengthen negative effect of religious struggle on well-being.

## Method

### Respondents and Procedure

The study included 143 adults, 81 women and 62 men, aged between 18 and 52 years. The mean age was 23.06 years (SD = 4.27). Most participants were single and had a secondary school education. All of them identified themselves as Roman Catholic. Table 1 presents demographic characteristics of the participants. Students in the third year of the undergraduate psychology program at the Catholic University of Lublin collected data through a web survey as a part of the course “Analysis and Interpretation of Empirical Data in Social Psychology,” which was taught by the first author in the 2017–2018 academic year. The procedure was approved by the Research Ethics Committee at the Institute of Psychology at the university where the study was conducted.

**Table 1:** Demographic characteristics of 143 respondents

Characteristic	*N*	%
Sex		
Female	81	56.6
Male	62	43.4
Education		
Secondary	113	79.0
Higher	30	21.0
Marital status		
Single	135	94.4
Married	8	5.6
Place of residence		
Village	46	32.2
City or town < 200,000 people	39	27.3
City > 200,000 people	58	40.6
Total	143	100.0

### Measurements

#### Religious and Spiritual Struggle Scale (RSSS)

The RSSS by Exline et al. (2014) was adapted to Polish by Zarzycka et al. (in press). The scale assesses six domains of religious and spiritual struggle: (1) Divine struggle involves negative emotions centered on beliefs about God or a perceived relationship with God; (2) demonic struggle involves concern that the devil or evil spirits are attacking an individual or causing negative events; (3) interpersonal struggle involves concern about negative experiences with religious people or institutions; (4) moral struggle involves wrestling with attempts to follow moral principles and feelings of worry or guilt about offenses the subjects perceive themselves to have committed; (5) religious doubt struggle involves feeling troubled by doubts or questions about one’s religious and spiritual beliefs; and (6) ultimate meaning struggle involves concern about not finding deep meaning in one’s life (Exline et al. 2014; Zarzycka et al., in press). The instructions included the following prompt:

Recall a difficult event or situation that you experienced in the last six months and that elicited references to God from you, e.g., you prayed to God, asked Him for help, expressed your resentment toward God, were angry at God, etc. Given this specific event, have you responded in each of these ways?

Then the participants rated 27 items of the RSSS using a 5-point Likert scale, from 1 (*not at all/does not apply*) to 5 (*a great deal*). The full scale and subscales were scored by averaging across items. Convergent and divergent validity of the individual subscales of the RSSS was confirmed (Zarzycka et al., in press). The internal consistency of the subscales of the RSSS obtained in this study is presented in Table 2.

**Table 2:** Descriptive statistics and bivariate correlations between the variables included in the study

Measure	1	2	3	4	5	6	7	8	9	10	11	12	13	14
1. Divine	–													
2. Demonic	.30***	–												
3. Moral	.42***	.45***	–											
4. Meaning	.45***	.21*	.30***	–										
5. Interpersonal	.24**	.30***	.22**	.29***	–									
6. Doubt	.49***	.30***	.42***	.51***	.45***	–								
7. Pure	.26**	.28**	.34***	.31***	.30***	.37***	–							
8. Identity	.27**	.31***	.28***	.28***	.32***	.41***	.67***	–						
9. Supportive	.26**	.23**	.34***	.25**	.31***	.35***	.74***	.75***	–					
10 Ruminative	.33***	.36***	.33***	.41***	.30***	.34***	.59***	.58***	.64***	–				
11. Confronting	.35***	.46***	.35***	.30***	.28***	.35***	.55***	.66***	.54***	.73***	–			
12. Social simulation	.22**	.09	.33***	.22**	.32***	.19*	.64***	.50***	.68***	.49***	.37***	–		
13. Perspective-taking	.25**	.33***	.26**	.24**	.31***	.29***	.60***	.72***	.73***	.63***	.65***	.52***	–	
14. Well-being	− .18*	− .17*	− .05	− .33***	.01	− .12	− .11	− .03	− .01	− .27**	− .20*	.05	− .10	–
*M*	1.90	1.89	2.58	2.49	2.02	2.41	2.83	3.01	2.87	2.51	2.57	3.52	2.57	3.70
SD	0.95	1.05	1.15	1.32	0.90	1.09	0.80	0.99	0.85	0.80	0.97	0.89	0.77	0.50
*α*	.85	.92	.91	.93	.80	.87	.67	.84	.81	.82	.82	.87	.70	.79

*p < .05, **p < .01, ***p < .001

#### Internal Dialogical Activity Scale (IDAS)

The IDAS by Oleś (2009) is based on Hermans’s dialogical self theory. It measures individual differences in an internal dialogical activity defined as engagement in dialogues with imagined figures, continuation or simulation of social dialogical relationships in one’s own thoughts, and confrontation of the points of view representing different “I-positions” relevant for personal or social identity (Puchalska-Wasyl et al. 2008). The IDAS contains 47 items, the first of which is a buffer item. Responses are rated on a 5-point Likert scale, from 1 (*I strongly disagree*) to 5 (*I strongly agree*). The IDAS assesses the general internal dialogical activity and seven types of IDs: (1) pure dialogical activity consists in conducting IDs spontaneously, thinking and resolving problems in a dialogical way; (2) identity dialogues involve IDs enhancing self-knowledge and answering identity questions, such as “Who I am?” and “What is important to me?”; (3) supportive dialogues involve IDs that are to confirm one’s own beliefs and to give support or instructions to oneself on the part of the imagined interlocutor; (4) ruminative dialogues involve IDs that delve into unpleasant topics and cause frustration, feelings of weariness and internal breakdown; (5) confronting dialogues involve IDs between two clearly separated parts of oneself, which consist in playing internal conflicts in the form of a dialogue; (6) social simulation dialogues involve mentally continuing or imagining dialogical social relations, quarrels, discussions, or exchange of ideas; (7) perspective-taking dialogues involve adopting a different point of view from one’s own, attempting to objectivize problems by viewing them from a new perspective (Oleś 2009).

The theoretical validity of IDAS was confirmed by Oleś (2009). Additionally, IDAS diagnostic validity was checked in two ways. The first way consisted in establishing the convergence of IDAS score with the declared frequency of internal dialogues. The second way consisted in checking the convergence of IDAS score with the numbers of inner voices and the relationship between them, which were identified by the means of phenomenological method of the circle. It was found that the IDAS score correlated positively with the number of inner voices (*r* = .41, *p* < .02); with the number of relationships between them (*r *= .48, *p* < .01); and with the number of supporting dialogues (*r* = .57, *p* < .01) (Oleś 2009). The internal consistency of the subscales of the IDAS obtained in the current study is presented in Table 2.

#### Psychological Well-Being Scale (PWBS)

The PWBS by Ryff (1989) consists of 18 items reflecting the six areas of psychological well-being: autonomy, environmental mastery, personal growth, positive relations with others, purpose in life, and self-acceptance. In the study, we used only the total score, which measures the overall well-being (Karaś et al. 2013). Respondents rated items on a scale from 1 (*strongly disagree*) to 6 (*strongly agree*). The Polish adaptation of the PWBS was carried out by Cieciuch (2011). The validity of 18-item PWBS was confirmed by using correlation of its subscales with subscales of 120-item version of the method (Ryff and Keyes 1995). The shortened subscales correlated from .70 to .89 with 120-item parent subscales. The internal consistency of the PWBS obtained in this study is presented in Table 2.

### Statistical Methods

**Fig. 1:**
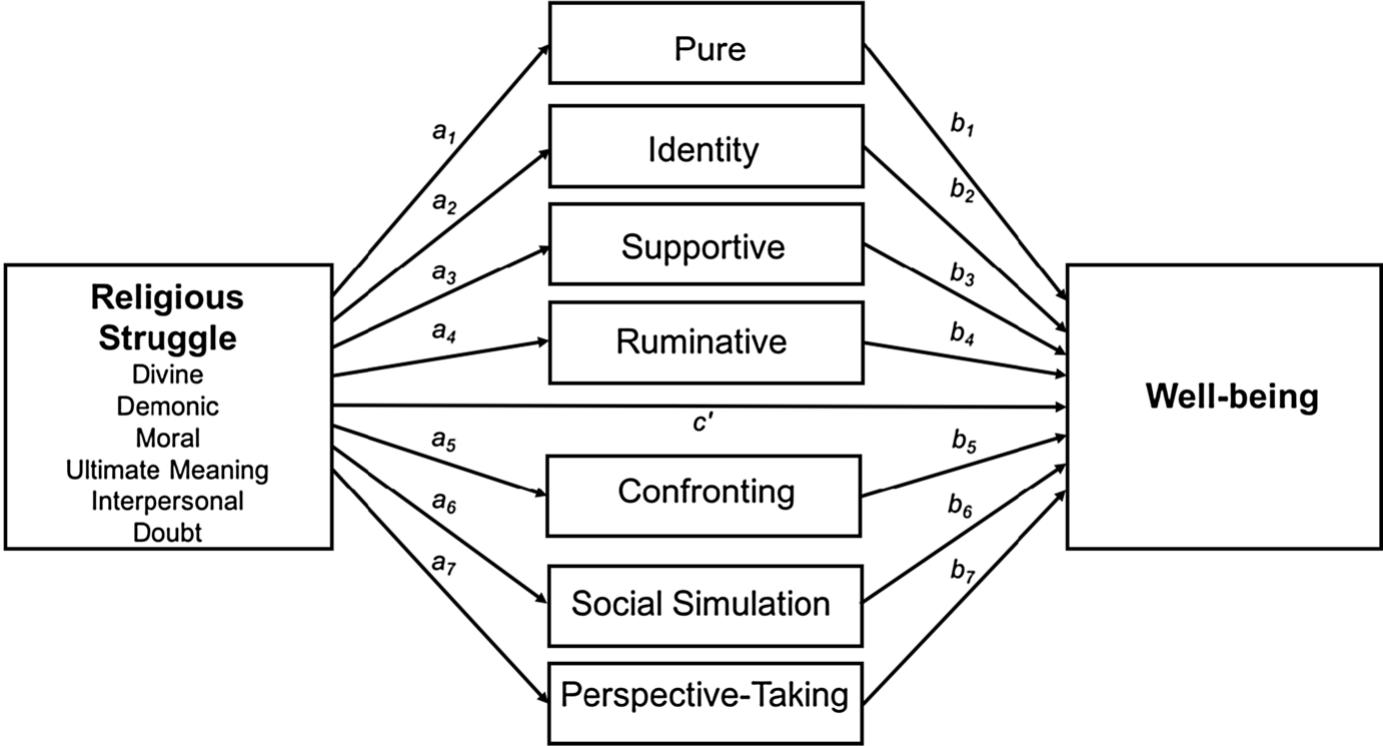
Diagram of the general mediation model. Note *c’*—direct effect of predictor on outcome while controlling for the mediator; *a*_1_, *a*_2_, *a*_3_, *a*_4_, *a*_5_, *a*_6_, *a*_7_—effect of the predictor on the mediator; *b*_1_, *b*_2_, *b*_3_, *b*_4_, *b*_5_, *b*_6_, *b*_7_—effect of the mediator on the outcome

This study was designed cross-sectionally. A series of correlational and regression mediation analyses was performed. First, we established whether there were correlations among the key constructs—religious struggle, internal dialogues, and well-being. To this end, we performed zero-order correlations among the PWBS and subscales of the RSSS and IDAS. In the regression models, divine, demonic, moral, ultimate meaning, interpersonal, and religious doubt struggles were examined as predictors of well-being. Furthermore, we examined whether these relationships might be mediated by seven forms of internal dialogues: pure dialogical activity, identity, supportive, ruminative, confronting, social simulation, and perspective-taking dialogues. Figure 1 shows the general mediation model.

We performed all mediation analyses using PROCESS (Hayes 2017), a regression-based path-analytic framework, and estimated indirect effects and bias-corrected confidence intervals. We tested the significance of indirect effects using the bootstrapping procedure. Unstandardized indirect effects were computed for each of 5000 bootstrapped samples, and the 95% confidence interval was computed. Prior to the main analyses, descriptive statistics were calculated for all variables in the study using SPSS v. 24.

## Results

### Basic Associations: Religious and Spiritual Struggle, Internal Dialogues, and Well-Being

We calculated Pearson bivariate correlations for all variables examined in the regression model. Results are shown in Table 2. All the types of religious/spiritual struggles had a significant positive correlation with the measured forms of IDs. The only exception was a nonsignificant correlation between demonic struggle and social simulation dialogues. Ruminative (*r *= − .27, *p* = .001) and confronting (*r* = − .20, *p* = .016) dialogues correlated negatively with well-being. The other forms of IDs did not correlate with well-being. Divine (*r *= − .18, *p* = .035), demonic (*r *= − .17, *p* = .044), and ultimate meaning struggle (*r *= − .33, *p* < .001) correlated negatively with well-being.

### Multiple Mediation Analyses: Religious and Spiritual Struggle–Internal Dialogues–Well-Being

The main analysis consisted of a series of regression mediation analyses that aimed to establish whether the seven forms of IDs—pure dialogical activity, identity, supportive, ruminative, confronting, social simulation, and perspective-taking dialogues—were mediators in the relationship between religious/spiritual struggles and well-being. We conducted six mediation analyses in which we tested divine, demonic, moral, ultimate meaning, interpersonal, and religious doubt struggle as predictors of well-being.

There were significant mediation effects of religious/spiritual struggle on psychological well-being through three types of IDs—ruminative, supportive, and social simulation dialogues. Table 3 shows outcomes of mediation analyses from religious struggle to well-being assessing indirect effects of ruminative, social simulation, and supportive dialogues, respectively.

**Table 3:** Outcomes of mediation analyses from religious struggle to well-being assessing indirect effects of internal dialogues

Model	*R*^2^	*c’*	*a*	*b*	*ab*	95% CI	
						Lower	Upper
Div–Rum–WB	.17^**^	.06	.28^***^	− .25^**^	− .07	− .124	− .032
Dem–Rum–WB	.16^**^	− .03	.27^***^	− .26^**^	− .07	− .124	− .035
Mor–Rum–WB	.15^**^	< .01	.23^***^	− .26^**^	− .06	− .107	− .029
Mean–Rum–WB	.21^***^	− .10^**^	.25^***^	− .20^*^	− .05	− .085	− .018
Inter–Rum–WB	.16^**^	.03	.27^***^	− .26^**^	− .07	− .135	− .029
Doubt–Rum–WB	.16^**^	− .03	.25^***^	− .25^**^	− .06	− .112	− .031
Div–Sim–WB	.17^**^	.06	.21^**^	.11	.02	.002	.063
Mor–Sim–WB	.15^**^	< .01	.25^***^	.10	.03	.001	.065
Mean–Sim–WB	.21^***^	− .10^**^	.14^**^	.11^*^	.02	.001	.047
Doubt–Sim–WB	.16^**^	− .03	.16^*^	.10	.02	.001	.050
Mor–Sup–WB	.15^**^	< .01	.25^***^	.15^^^	.04	.002	.087
Mean–Sup–WB	.21^***^	− .10^**^	.16^**^	.13	.02	.001	.057
Inter–Sup–WB	.16^**^	.03	.30^***^	.15^^^	.04	.003	.107
Doubt–Sup–WB	.16^**^	− .03	.27^***^	.15^^^	.04	.002	.091

Div = divine; Dem = demonic; Mor = moral; Mean = ultimate meaning; Inter = interpersonal; Doubt = religious doubt; Rum = ruminative; Sim = social simulation dialogues; Sup = supportive dialogues; WB = well-being; *c’* = direct effect of predictor on outcome while controlling for the mediator; *a* = effect of the predictor on the mediator; *b* = effect of the mediator on the outcome; *ab* = indirect effect of predictor on outcome through the mediator; *R*^*2*^ = amount of variance explained by the model; CI = confidence intervals

^^^*p* < .10, *p < .05, **p < .01; ***p < .001

These mediations differ from each other in that ruminative dialogues cause religious/spiritual struggles to decrease well-being (all indirect effects are negative, *ab* = from − .05 to − .07), while supportive and social simulation dialogues cause the same struggles to strengthen well-being (all indirect effects are positive, for supportive dialogues *ab* = from .02 to .04 and for social simulation *ab* = from .02 to .03).

The indirect effects of ruminative dialogues in the relationships between religious/spiritual struggles and well-being are non-specific: each of the measured types of religious/spiritual struggles strengthens the tendency to conduct ruminative dialogues (*a* = from .23 to .28), which in turn reduces well-being (*b* = from − .20 to − .26).

The indirect effects of supportive dialogues and social simulation dialogues in the relationships between religious/spiritual struggles and well-being show some variation. The intrapersonal forms of struggle—moral, ultimate meaning, and religious doubt struggle—increase well-being both through supportive dialogues (*ab* = from .02 to .04) and social simulation dialogues (*ab* = from .02 to .03). This means that people who have high scores in intrapersonal struggles are more likely to conduct supportive dialogues (*a *= from .16 to .27) and social simulations dialogue (*a *= from .14 to .25), which in turn strengthens their well-being (for supportive dialogues *b *= from .13 to .15 and for social simulation dialogues *b *= from .10 to .11). In addition, social simulation dialogue was an important mediator in the relationship between divine struggle and well-being (*ab* = .02), while supportive dialogues mediated the relationship between interpersonal struggle and well-being (*ab* = .04).

While controlling for the mediators, the direct effects of religious/spiritual struggles on well-being were not significant for all types of struggles excluding ultimate meaning; thus, these were full mediations. In the case of ultimate meaning struggle the mediations were partial. It should be emphasized that ultimate meaning struggle directly decreases well-being (*c’* = − .10); however, combining this type of struggle with supportive or social simulation dialogues increases well-being.

## Discussion

In the study, we aimed to explore relationships between religious/spiritual struggle, internal dialogical activity, and well-being. Although a large number of studies suggest that this type of struggle worsens health and well-being (e.g., Abu-Raiya et al. 2015; Ellison and Lee 2010; Ellison et al. 2010; Exline and Rose 2013; Fitchett et al. 2004; McConnell et al. 2006), some researchers have claimed that people can benefit from their struggle in the religious or spiritual realm (Desai 2006, 2015; Pargament et al. 2006; Zarzycka and Zietek 2018). The latter studies indicate that mediators and moderators play important roles in predicting well-being during religious/spiritual struggle (Ellison et al. 2013; Zarzycka et al. 2017; Zarzycka and Zietek 2018). The results we obtained show that internal dialogical activity is a mediator of the relationship between religious/spiritual struggle and psychological well-being. Depending on what types of IDs a person conducts during their struggles, this can result in an increase or decrease in well-being.

One finding worth emphasizing is that religious/spiritual struggle is clearly related to internal dialogical activity—with one exception (nonsignificant correlation between demonic struggle and social simulation dialogues) each of the measured types of struggle coincides with each type of ID. Why? It seems that religious struggle is associated with being occupied with uncertainty, which is one of the typical experiences that trigger an ID. Hermans and Hermans-Konopka (2010) claim that the experience of uncertainty is composed of four aspects: (a) *complexity*, which refers to many parts (of self and society) that have a variety of interconnections; (b) *ambiguity*, pertaining to a suspension of clarity, as the meaning of one part is determined by the flux and variation of the other parts; (c) *deficit knowledge*, concerning the absence of a superordinate knowledge structure that is able to resolve contradictions between the parts; and (d) *unpredictability*, implying a lack of control of future developments. These aspects of uncertainty are inherent in the religious/spiritual struggle in many ways. Much as religious/spiritual struggle might take the form of a single, primary negative emotion (e.g., sadness, anger, guilt), it usually represents a conflict in which people experience complex thoughts or feelings that they cannot easily reconcile (complexity). There is an ambiguity in people’s relation to God, who is a relational partner with unique qualities: all powerful, all knowing, and holy. Most people report that they cannot see or hear God with their physical senses. This lack of unambiguous sensory evidence (deficit knowledge) prompts many questions about God’s qualities, communication, and very existence (Exline 2013). Finally, when people try to rely on their religious faith, but they have the feeling that God does not respond to their prayers, religion ceases to be “a safe home.” Then uncertainty appears regarding whether God really exists and cares about the person’s life (unpredictability). Religious doubts and incompatibilities between personal beliefs and the content of the religious doctrine result in the weakening of the individual’s worldview in such a way that it ceases to fulfill a unifying philosophy of life (Allport 1950). According to Hermans and Hermans-Konopka (2010), the experience of uncertainty can be seen, on the one hand, as a situation that opens a broad range of unexpected possibilities; on the other hand, too high a level of intensity of uncertainty can lead to anxiety and insecurity. The researchers argue that ID is one of the forms of reduction of the experience of uncertainty.

Puchalska-Wasyl and Oleś (2013) agree that IDs can reduce uncertainty. At the same time, they stress that such effect is neither universal nor obvious. They are of the opinion that if a person is unable to unify or integrate various viewpoints emerging as a starting point for an ID, or none of the viewpoints is able to “convince” the others, the doubts even increase. Presumably, this situation appears when religious/spiritual struggle is accompanied by ruminative IDs. Such IDs cause frustration, feelings of weariness, and internal breakdown rather than integration, and consequently, as our study showed, they worsen people’s well-being during religious/spiritual struggle. It is worth highlighting that the effect mentioned above was visible in the case of all measured types of religious/spiritual struggles. This means that people tend to ruminate during their struggle, which has a detrimental effect on their well-being. This is in line with the view of “the cumulative stress effect.” If religion stops working as a personal resource—if, on the contrary, it generates stress—then stress accumulates (cf. Hobfoll 2001) and impairs mental health (Zarzycka 2017).

Apart from the negative impact of ruminative IDs on well-being, we have observed that two types of IDs, supportive and social simulation dialogues, can have a positive function and result in an increase in well-being when religion is the source of strain. These findings can be explained in the context of the opinion that the experience of uncertainty is reduced by an ID because the encounter of two or more points of view in dialogue allows for clarifying or overcoming uncertainty either by “integration of opposites” and creation of a new solution or by making one viewpoint stronger than the other (Hermans and Hermans-Konopka 2010; Puchalska-Wasyl and Oleś 2013). The function of supportive dialogues is reflected in their name. They aim to confirm one’s own beliefs, give support or instructions to oneself; thus, by definition they serve to enhance one viewpoint and, as a consequence, they provide confidence and reduce (to some degree) uncertainty, which can increase well-being. The mechanism of raising well-being by social simulation dialogues seems to be less obvious because functions of such IDs can be more diverse. This type of ID is understood as mentally continuing or imagining dialogical social relations, quarrels, discussions, or exchange of ideas; thus, they are defined by the interlocutor representing a person from the social environment, not by the function. Taking into account seven key functions of IDs identified by Puchalska-Wasyl (2006, 2016a, c), it seems probable that social simulation dialogues can fulfill all of them, namely support, substitution, exploration, bond, self-improvement, insight, and self-guiding (see introduction). Further research is needed in order to answer what functions of social simulation dialogues are engaged in enhancing well-being during religious struggle.

It is worth mentioning that intrapersonal forms of struggle—that is, moral, ultimate meaning, and religious doubt struggle—increase well-being through both supportive and social simulation dialogues. However, divine struggle can lead to strengthening well-being only through social simulation dialogues, whereas interpersonal struggle increases well-being only through supportive dialogues. One can conclude from the above that when people experience negative emotions toward God (divine struggle) they do not seek simple support, but rather contact (even imagined) with a person who does not reject (bond), but helps to get a distance (insight), shows mistakes or warns against the further errors (self-improvement), outlines new possibilities (exploration, self-guiding), and motivates to take action (self-guiding). It is possible that during a divine struggle a man is looking for contact with an authority. An analogy here is provided by studies on absolving the guilt of Catholics and Protestants. The Catholic theology states that guilt is present, but that it can be resolved. The Catholic Church offers guilt-absolving rituals, for example a service in which people can confess their guilt to God through the agency of a special person (a priest). In Protestant teaching, guilt is not dealt with in that way. The research suggests that Catholics show more constructive guilt reaction than Protestants (Walinga et al. 2005) and the perception of grace or unconditional divine forgiveness better mitigates Catholics’ feelings of guilt (Watson et al. 1988). In turn, when a person has negative experiences with religious people or institutions (interpersonal struggle), first of all, he or she is looking for support. By means of supportive IDs, the person can get it not only from another imagined person, but also from another part of their self (e.g., I as a believer, I as an optimist, or I as understanding person).

In regards to shortcomings of the study, it should be emphasized that the cross-sectional, non-experimental design limits our ability to make causal interpretation about the findings. Next, the study was based on individuals’ self-reports, and thus, the response bias could not be controlled. However, this possibility may be tempered somewhat by the fact that respondents completed the measures anonymously. Further, the sample size can be considered relatively small. It consisted of adults from one country; in fact it included only Polish Roman Catholics. Therefore, the results need replication with larger samples and inclusion of people of different faiths. It would be also advisable to go deeper than just denominational labels. Finally, the obtained mediation effects were not very high which also suggests that they need to be examined in much more depth.

Taken together, this study aims to explain the mechanisms of the influence of religious/spiritual struggles on well-being. In light of our findings, one can say that religious struggle, regardless of which religious matters the struggle refers to, triggers IDs. Internal dialogical activity serves as a mediator of the relationship between religious/spiritual struggle and psychological well-being. Depending on the types of IDs people conduct during their struggles, this can result in an increase or decrease of well-being. Ruminative dialogues enhance the detrimental effect of religious struggle on well-being, while social simulation and supportive dialogues make religious/spiritual struggle lead to positive outcomes. The results should be replicated in studies in which the shortcomings of the current study will be minimized.
